# Differential Expression of MicroRNAs During Root Formation in *Taxus Chinensis* Var. *mairei* Cultivars

**DOI:** 10.1515/biol-2019-0011

**Published:** 2019-04-06

**Authors:** Yongjun Fei, Caroline Luo, Wei Tang

**Affiliations:** 1 College of Horticulture and Gardening, Yangtze University, Jingzhou, Hubei Province 434025, Jingzhou, People’s Republic of China; 2 Department of Microbiology, University of North Carolina at Chapel Hill, Chapel Hill, NC 27599, Chapel Hill, USA

**Keywords:** Conifer, Nutrition, Root formation, Taxus

## Abstract

MicroRNAs (miRNAs) have been shown to play key roles in the regulation of plant growth and development by modifying the expression of their target genes. However, the influence of miRNAs on root formation and development in woody plants, such as *Taxus chinensis*, remains largely unknown. In the current study, we explored the phytohormone-response and nutrition-response miRNA expression profiles during *T. chinensis* rooting by quantitative real-time PCR (qPCR). We identified six phytohormone-response miRNAs, namely, miR164a, miR165, miR167a, miR171b, miR319, and miR391, and eight nutrition-response miRNAs, namely, miR169b, miR395a, miR399c, miR408, miR826, miR827, miR857, and miR2111a, that were differentially expressed at different rooting phases of *T. chinensis*. Using northern blot analysis of the putative target genes of these miRNAs, we detected the relative gene expression changes of the target genes. Taken together, our results suggest that miRNAs are involved in root formation of *T. chinensis* and that miRNAs may play important regulatory roles in primary root, crown root, and root hair formation by targeting phytohormone and/or nutrition response genes in *T. chinensis*. For the first time, these results expand our understanding of the molecular mechanisms of plant root formation and development in a conifer species.

## 1 Introduction

MicroRNAs (miRNAs) are short non-coding RNAs that regulate many signaling pathways and metabolic mechanisms by repressing the expression of their target genes [1, 2, 3]. MiRNA-mediated gene regulation and signaling mechanisms have been shown to be very important for plant growth and development [4, 5, 6]. Thousands of miRNAs have been identified, including many well-studied miRNAs, such as miR156 and miR399, which are highly conserved among different plant species [2, 7, 8]. The expression of miR164a, miR165, miR319, miR167a, miR171b, and miR391 has been previously reported to be an important regulator of root growth and development [9, 10, 11]. The targets of these miRNAs include auxin-response genes, such as ARF6, which is the target of miR164a, ARF6 and ARF8, which are the targets of miR167a, and ARF10, which is the target of miR165 and miR319. ARF6 and ARF16 are the targets of miR171b. ARF16 and ARF17 are the targets of miR391 [12, 13, 14, 15].

Although research studies have been performed in model plants [16, 17], knowledge of miRNA regulation in woody plant root growth and development is limited, and this regulation is far from being fully understood. *Taxus chinensis* (Chinese Yew) is well known for its high value in the treatment of various cancers [18, 19, 20, 21]. Investigating the molecular and genetic regulatory mechanisms of root development in *T. chinensis* is important for the development of new varieties. Understanding the role of these miRNAs could enable the development of novel biotechnology approaches to genetically engineer plants with improved plant growth, nutrient uptake, and productivity. In plants, the root is established from cells located on the root apical meristem [22, 23]. Transcription factors, auxin-response factors, and nutrition response genes contribute to root initiation, growth, and development [24, 25, 26]. For example, the transcription factors *PLT*, *SHR*, and *SCR* regulate the maintenance of the root meristem in *Arabidopsis* [5, 27, 28]. Homologs of these genes have been identified in many plant species, including monocots, dicots, and woody plants [5, 22, 29]. In rice, *OsWOX3A* has been reported to regulate lateral root initiation and root hair development, and *OsSCR1* is involved in the formation, growth, and development of the primary root [23, 30, 31]. Phytohormones and nutrition have been reported to play important roles in the regulation of root formation [32, 33, 34]. For example, auxin-response genes regulate the expression of the transcription factor gene *CRL1*, resulting in a reduced lateral root number and lateral root formation [16, 22]. The expression of the root formation-related genes *LBD16* and *LBD29* is regulated by ARF7 and ARF19. The expression of ARF7 and ARF19 controls the development of the lateral root in plants [15]. The knockdown of *OsARF16* results in reduced sensitivity in primary roots, lateral roots and root hairs towards auxin [9, 13, 35, 36].

Nutrients influence root development, including root length, diameter, lateral root branching, and root angle [32, 33, 34]. Phosphate (Pi) deficiency has been reported to result in a decreased primary root length and increased lateral root density in *Arabidopsis* [33, 37, 38]. In rice, Pi deficiency leads to an increased primary root length and redistribution of the auxin content [38, 39]. Nitrogen (N) nutrition plays important roles in the development of the root in numerous plant species [40, 41, 42]. In *Arabidopsis*, the elongation of both the primary root and lateral root is reduced when the nitrate availability is increased [4, 32, 40]. In addition, environmental stimuli, including drought stress, high-salt stress, and low temperature stress, influence root development by multiple signaling transduction pathways [2, 7, 23]. Numerous stress-related transcription factors have been reported to be important for root development [23, 43, 44]. For example, the overexpression of *OsNAC10* and *OsNAC5* in a root-specific manner leads to increased root growth, and the overexpression of the aspartic protease gene *ASPG1* results in a decrease in water loss [24, 45, 46].

In the current study, we profiled the expression of phytohormone-response and nutrition-response miRNA in *T. chinensis* shoots during different phases of the rooting process and simultaneously analyzed their target gene expression to uncover the miRNA regulatory roles in root growth and development in *T. chinensis*. We identified both increased and decreased miRNA expression levels and upregulated and downregulated expression of target genes during rooting. Our results indicate that these phytohormone-response and nutrition-response miRNAs play potentially important roles in *T. chinensis* root formation.

## 2 Materials and Methods

### 2.1 Plant materials

Shoots of *Taxus chinensis* var. *mairei* cultivars were harvested from the Tree Breeding Program of *T. chinensis*. The shoots were thoroughly cleaned by following previously described procedures [47] before use for rooting. In brief, shoots of *T. chinensis* were collected at the appropriate size of 10-15 cm. The cut ends of the shoots were washed and rinsed with sterile distilled water for 10 minutes. After washing, the cut ends were air dried on filter paper and placed under diffused light (30 μmol m^−2^s^−1^ PPFD) for 20 minutes. The dried shoots were shifted to normal light for another 20 minutes and then transferred to containers containing an autoclaved mixture of perlite, peat moss, and vermiculite at a ratio of 1:1:1 v/v/v in a greenhouse for in vitro rooting.

### 2.2 In vitro rooting and determination of rooting phases

**Figure 1:**
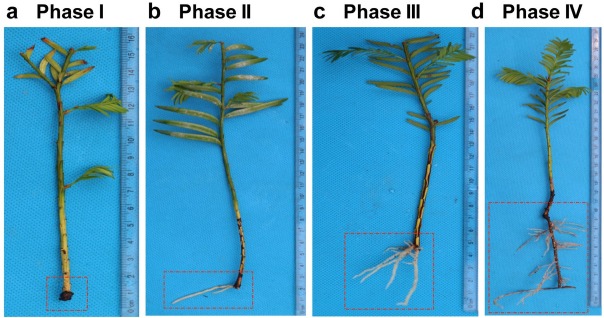
**The phases of in vitro rooting in *Taxus chinesis***. Rooting process was divided into four phases. (a) Phase I: at 21 days of in vitro culture, no root formation. (b) Phase II: at 42 days of culture, one primary root and 1-3 crown roots, but without lateral roots. (c) Phase III: at 63 days of culture, shoots with primary root, more than 3 crown roots, and lateral roots. (d) Phase IV: at 84 days of culture, shoots with primary root that grows more than 5 cm and with lateral roots and root hairs.

Clean shoots were transferred to containers containing a perlite:peat moss:vermiculite (1:1:1 v/v/v) mixture and grown in a greenhouse until the root tissues were collected for different assays. Rooting was maintained at 25°C in the light (50 μmol m^-2^s^-1^, 16-h photoperiod). Rooting was monitored weekly at the same time. Each Monday, we examined rooting by randomly selecting 30 shoots from the containers. If no roots were observed, the shoots were returned to the containers for rooting. If roots were observed, we recorded the number of roots and then returned the shoots to the containers for continued growth. Each week, we examined different containers. During the 12-week examination period, we observed obvious changes in the rooting status on the 21st, 42nd, 63rd, and 84th days of culture. Based on the changes, we divided rooting into four phases for the miRNA expression analysis. The phases of rooting were determined based on the degree of root formation and growth. On the 21st day of rooting, no roots were observed from the shoots, and this stage was defined as rooting Phase I. The tissues used for the RNA extraction were obtained from the basal portion of the cuttings as indicated in the red frame shown in Fig. 1. The basal portion of the cuttings includes the root primordium and callus. Rooting Phase II was defined as the stage during which the shoots had a primary root and 1-3 crown roots without lateral roots. We found that 42 days were required for the shoots to reach rooting phase

II. On the 63rd day of rooting, the shoots grew with a primary root, more than 3 crown roots, and lateral roots. We defined this rooting stage as Rooting Phase III. Rooting Phase IV was defined as the stage characterized by shoots with a primary root that grew more than 5 cm with lateral roots and root hairs. The period from the first day of rooting to rooting Phase IV is 84 days. The rooting experiment was repeated three times. Three hundred shoots were used each time. In each replicate, we obtained similar results. The rooting assay is highly reproducible. In total, 1080 shoots were transferred to containers, and 360 shoots were ultimately used in the rooting assays.

### 2.3 MicroRNA profiling by quantitative real-time PCR (qPCR)

The total RNA was extracted from rooting tissues at different phases using TRIzol reagent following the manufacturer’s protocol (Invitrogen, CA). Then, the total RNA was assayed with a Nanodrop (Thermo Fisher Scientific, CA) to determine the quantity and quality. For the miRNA expression analysis, a TaqMan® MicroRNA Reverse Transcription Kit (Applied Biosystems Inc., CA) was used to prepare high-quality cDNA. The qPCR reactions for miRNAs were carried out with TaqMan primers and probe sets from ABI (Applied Biosystems Inc., CA). The PCR reactions were initiated by incubation at 50°C for 2 minutes and 95°C for 10 minutes, followed by 40 cycles at 95°C for 15 sec and 60°C for 60 sec. The U6 snRNA was chosen as the endogenous control.

The delta–delta Ct method was used to obtain the expression values. Samples in triplicate were examined on an Applied Biosystems 7900HT System following the manufacturer’s instructions. The primers used in this study are listed in Table 1. The comparative threshold cycle method was applied to calculate the relative gene expression difference among the rooting groups at different phases. We did not analyze the expression of microRNAs and the targets of these microRNAs in different types of roots.

**Table 1:** Primers used in this study

Primer name	Primer sequence (5’-3’)
ARF6-F	CAAAGTTTAGCAGCTACCACGA
ARF6-R	ACGTCGTTCTCTCGGTCACGAC
ARF8-F	TTTGCTATCGAAGGGTTGCGTTG
ARF8-R	CATGGGTCATCACCAAGCGGA
ARF10-F	GGTTTCTCCGTTCCACCGGTTATT
ARF10-R	CCGTGGATGTCTTTAGCAATCA
ARF16-F	CGTTAAGCTCTGTTCTGGCGAC
ARF16-R	AGTAATGGTGAAGATCCCGGAAG
ARF17-F	GCACCTGATCCAAGTCCTCGTC
ARF17-R	GGTGAATAGCTGGGGAGCGGAT
LAC3-F	TCGCTTTCCTCGCTTCTGCCGTGA
LAC3-R	ACCACAAGCGTTGGACCACGGGGT
LAC12-F	AGAGACGCCGGTGAAGCGAGGCT
LAC12-R	CTTCGAGCGTAGGCCCCCGGGA
AOP2-F	AGAGGACAAGATACACAGCGCAGCA
AOP2-R	AAGTCGCGGTAATCAAAACGGGTC
NLA-F	ACAATTGTTCTCGTGAATGCCGCC
NLA-R	GAGCATGCTCGTTAAACCACGTCC
PHO2-F	CCCCTTTGAAGTTTATCCAACGCTGG
PHO2-R	AGGTGAGCCAACTGAGGACTCGCC
miR164a-F	CACCCACTTTCGACCCTTAAACTCGCTCCA
miR164a-R	TGAAGCTAGGAAAGAGGAGCTCGTTG
miR164c-F	CACCTCAGGCTTCTTTAATTCGCGTGGTG
miR164c-R	AACTTAGACTGTGCAAAGCCCGAAA
miR165-F	CACCTGAATGAAACTGTCCAACGACACA
miR165-R	CGTCGCTAGCTACCAACAACGA
miR167a-F	GCAGCCTGAAGCTGCCAGCGCAT
miR167a-R	GTCGTATCCAGTGCAGGGTCCGAGGTATTCGCACTGCGCGACTAGATC
miR170-F	GCATGCTGCCTGGCTCCCTCGGT
miR170-R	GTCGTATCCAGTGCAGGGTCCGAGGTATTCGCACTGGCGCGACTGGCATA
miR171b-F	GCGGCGAGAATCTTGATCGGATG
miR171b-R	GTCGTATCCAGTGCAGGGTCCGAGGTATTCGCACTGGATACGCATGCAG
miR319-F	CGGGATCCTGGAATTCCGGGACGCCC
miR319-R	GGGGTACCAAGCCACAGAAGTCAACCGACA
miR391-F	GCGTCGACTTTGGGGCCCTCACCGAAC
miR391-R	GGGGTACCCGGAATTCCATGCTCGCTGC
miR824-F	GACGCTACGAGCCACTTGCGAA
miR824-R	GTCGTATCCAGTGCAGGGTCCGAGGTATTCGCACTGGATACGACTTCGGT
miR169b-RT	GTCGTATCCAGTGCAGGGTCCGAGGTATTCGCACTGGATACGACCCCGGCA
miR169b-F	GCAGCCAGCCAAGGATGACCGT
miR395a-RT	GTCGTATCCAGTGCAGGGTCCGAGGTATTCGCACTGGATACGACGCGGTTC
miR395a-F	GCACGTCTGAAGTGTTTGGGCGG
miR398b-RT	GTCGTATCCAGTGCAGGGTCCGAGGTATTCGCACTGGATACGACGCGGTGT
miR398b-F	GCAGCGAGGGTTGATATGAGCGA
miR399c-RT	GTCGTATCCAGTGCAGGGTCCGAGGTATTCGCACTGGATACGACCAGGCGGC
miR399c-F	GCGACGTGCCAAAGGAGAGTCGT
miR408-RT	GTCGTATCCAGTGCAGGGTCCGAGGTATTCGCACTGGATACGACCATGCGCT
miR408-F	GTCAGCACAGGGAACAAGCACGG
miR826-RT	GTCGTATCCAGTGCAGGGTCCGAGGTATTCGCACTGGATACGCGACGTA
miR826-F	GCAGCCTAGTCCGGTTTTGGACG
miR827-RT	GTCGTATCCAGTGCAGGGTCCGAGGTATTCGCACTGGATACGACACGTTG
miR827-F	GGCGCGUUAGAUGACCAUCAACG
miR857-RT	GTCGTATCCAGTGCAGGGTCCGAGGTATTCGCACTGGATACGACACGACAC
miR857-F	GCGGCGTTTTGTATGTTGAAGCG
miR2111-RT	GTCGTATCCAGTGCAGGGTCCGAGGTATTCGCACTGGATACGACTACGCC
miR2111-F	GGCAGCTAATCTGCATCCTGACG

### 2.4 Determination of miRNAs for microRNA profiling

Based on previous publications [48, 49, 50], we selected nine hormone-response microRNAs, including miR164a, miR164c, miR165, miR167a, miR170, miR171b, miR319, miR391, and miR824. These miRNAs have been shown to regulate root formation in non-woody plants [48, 49, 50]. MiR164a regulates lateral root development by targeting

ARF6 and ARF16 in *Arabidopsis* [50], miR164c regulates lateral root formation by targeting ARF17 in *Zea mays* [14, 24, 48, 51, 52], miR165 modulates shoot apical meristem development in *Arabidopsis*, rice, and maize [14, 24, 48, 51, 52], miR167a regulates root and pollen development by targeting ARF6 and ARF8 [2, 13, 48, 49, 53, 54, 55, 56, 57], miR170 regulates root indeterminacy, miR171b participates in the maintenance of shoot and root indeterminacy [24, 41, 54, 58], miR319 regulates leaf development by targeting TCP [1, 56, 59], miR391 regulates leaf development and auxin response, and miR824 regulates flower development by targeting ARF10 [36, 60, 61, 62].

Additionally, we selected nine nutrition-response microRNAs that have been demonstrated to regulate root formation in non-woody plants, including miR169b, miR395a, miR398b, miR399c, miR408, miR826, miR827, miR857, and miR2111a. Among these miRNAs, miR169b regulates nitrogen homeostasis [24, 59, 63, 64, 65], miR395a regulates sulfate uptake and translocation [43, 44, 59, 66, 67, 68], miR398b regulates the copper starvation response [2, 24, 44, 64], miR399c regulates Pi uptake and translocation by targeting PHO2 [37, 38, 39, 43, 44, 69, 70, 71], miR408 regulates the copper starvation response and copper homeostasis by targeting LAC3 and LAC12 [24, 48, 59, 64, 72, 73], miR826 regulates the nitrogen starvation response by targeting AOP2 [74], miR827 regulates nutrient recycling and Pi uptake and translocation by targeting NLA [24, 72], miR857 regulates the copper starvation response and copper homeostasis [72], and miR2111a regulates the phosphate starvation response [70]. To determine the functions of these miRNAs, we profiled the expression of these 18 miRNAs by qPCR and examined their target gene expression through northern blotting.

### 2.5 Northern blot analysis

The total RNA was extracted from rooting tissues at different phases using TRIzol reagent following the manufacturer’s protocol (Invitrogen, CA). Northern blotting was carried out as previously described [75]. The quality of the RNA was checked by a Nanodrop and gel electrophoresis. The concentration of RNA was measured using a Nanodrop. The equal loading of the RNA samples was verified by EB staining. Each time, 5 μg RNA per sample were used for blotting. rRNA was used as a loading control. GAPDH RNA was used as a control for comparison. The baked blots were pre-hybridized in 1 M NaCl, 1 % SDS, 10% dextran sulphate and 50 μg/ml denatured herring sperm DNA at 65°C, rinsed with 0.19 SSPE (1 × SSPE contains 180 mM NaCl, 10 mM NaH^2^PO^4^, and 1 mM EDTA, pH 6.5) and 0.5% SDS at 45°C. The hybridization probes were labeled with γ-[^32^P]-ATP and used to detect their gene expression level. Five biological replicates were used. The images of the northern blots were taken using a UVP BioSpectrum imaging system, and Image Analysis Software was used to determine the *density* of each band. GraphPad Prism 6 (GraphPad Software Inc., CA) was used to analyze the data.

### 2.6 Statistical analyses

The mean values were used to determine the significant differences among the different phases with the Least Significant Difference test at a 5% level of probability. The statistical analysis of the data was performed by Student’s t-test or one-way ANOVA using GraphPad Prism 6 (GraphPad Software Inc., CA).

## 3 Results

### 3.1 Phases of rooting in *T. chinensis*

First, we divided the rooting process into four phases to collect root tissues during each phase and obtain a good dynamic view of the miRNA involvement during root formation in *T. chinensis*. The detailed rooting processes are described in the Materials and Methods.

### 3.2 Profiling of phytohormone-response miRNAs during *T. chinensis* root formation

**Figure 2:**
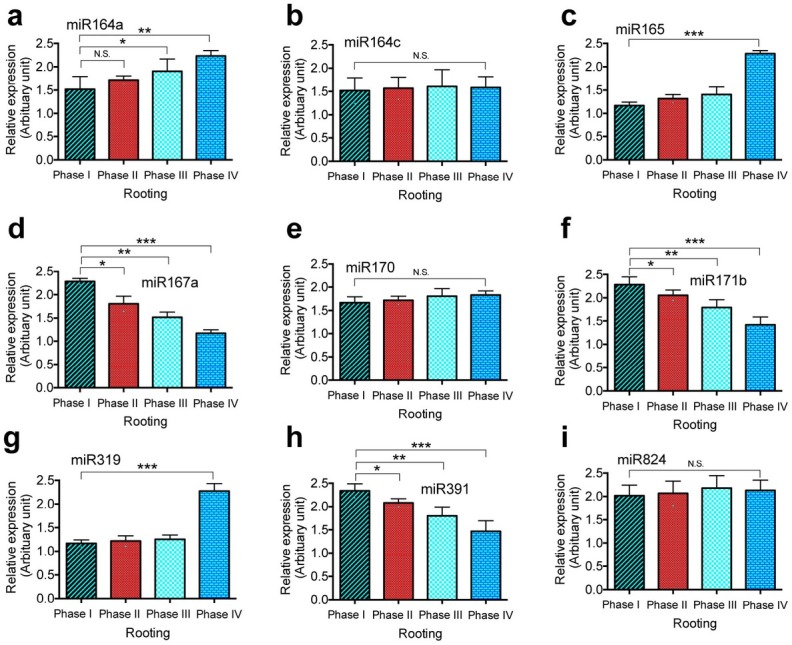
**The involvement of hormone-response miRNAs in root formation in *Taxus chinesis***. Nine miRNAs were profiled during in vitro rooting process. These miRNAs include miR164a (a), miR164c (b), miR165 (c), miR167a (d), miR170 (e), miR171b (f), miR319 (g), miR391 (h), and miR824 (i). Total RNA samples were prepared from root tissues of *T. chinensis* harvested at indicated time points and used for qPCR. The *U6* gene was used as an internal reference gene control. Experiment was repeated three times with three biological repeats. Statistically significant differences between groups were determined by one-way ANOVA. Data are presented as means of three independent experiments. Vertical bars indicate standard error. Asterisks indicate significant differences compared to Phase I, as assessed by a *t*-test. *P<0.05, **P<0.01, ***P<0.001, N.S., not statistically significant.

To examine the involvement of miRNAs in the rooting process in *T. chinensis*, we profiled the expression of nine hormone-response miRNAs by performing quantitative real time PCR (qPCR). The nine phytohormone-response/ related miRNAs examined included miR164a (Fig. 2a), miR164c (Fig. 2b), miR165 (Fig. 2c), miR167a (Fig. 2d), miR170 (Fig. 2e), miR171b (Fig. 2f), miR319 (Fig. 2g), miR391 (Fig. 2h), and miR824 (Fig. 2i). The total RNAs were extracted from the roots at each time point during the four phases of the root formation process. The *U6* gene was used as an internal control. Our results demonstrated that during rooting in vitro, the expression levels of the auxin response-related miR164a, miR165, and miR319 were significantly increased (Fig. 2). The expression level of miR164a was increased from phase II through phase IV (Fig. 2a). The expression levels of miR165 and miR319 were increased at rooting phase IV (Fig. 2c and 2g). Meanwhile, our results showed that the expression levels of miR167a, miR171b, and miR391 were significantly decreased compared with those in the non-rooted shoots (Fig. 2). The expression levels of these three miRNAs were decreased the most by rooting phase IV (Fig. 2d, 2f, 2h). In addition, we found that the expression levels of miR164c (Fig. 2b), miR170 (Fig. 2e), and miR824 (Fig. 2i) did not change throughout the four phases of the rooting process. Because our examination focused on the rooting phases and not the root types, some of the total changes in expression could be due to the tissue type used in the analysis.

#### 3.2.1 Expression of phytohormone-response miRNA target genes in *T. chinensis* roots

**Figure 3:**
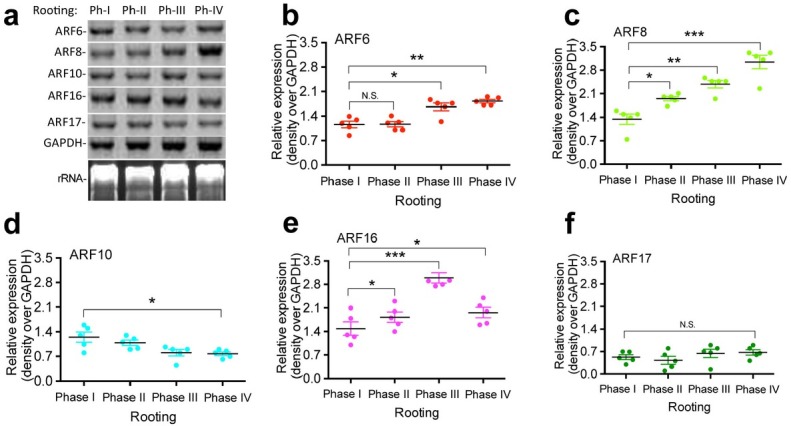
**Expression of hormone-response miRNA target genes in *T. chinensis* root tissues at different root formation and developmental stages**. Total RNA samples were prepared from root tissues of *T. chinensis* harvested at different rooting phases and used for northern blotting analysis (a). Antisense oligonucleotides of *ARF6* (b), *ARF8* (c), *ARF10* (d), *ARF16* (e), and *ARF17* (f) were labeled with γ–[^32^P]ATP and used as probes to detect their transcript level. GAPDH rRNA was used as a loading control. Statistically significant differences between groups were determined by one-way ANOVA. Data are presented as means of five independent experiments. Vertical bars indicate standard error. Asterisks indicate significant differences compared to Phase I, as assessed by a *t*-test. *P<0.05, **P<0.01, ***P<0.001, N.S., not statistically significant.

In addition to profiling the expression of the phytohormone-response miRNAs, we also investigated the expression of the target genes of the nine phytohormone-response miRNAs in *T. chinensis* roots at different rooting phases. We particularly focused on using a northern blot analysis (Fig. 3a) to examine the expression of the putative target genes *ARF6* (Fig. 3b), *ARF8* (Fig. 3c), *ARF10* (Fig. 3d), *ARF16* (Fig. 3e), and *ARF17* (Fig. 3f) in the roots at different rooting phases. The total RNA samples were prepared from root tissues of *T. chinensis* harvested at different rooting phases, and a northern blot analysis was performed (Fig. 3a). Our results showed that the expression of *ARF6* (Fig. 3b) and *ARF8* (Fig. 3c) was strongly induced throughout root formation and that the expression of *ARF16* (Fig. 3e) was increased during rooting phase III. By contrast, the expression of *ARF10* (Fig. 3d) was reduced, and the expression of *ARF17* (Fig. 3f) remained very low throughout the entire process of root formation. Our analysis on the expression of the putative target genes *ARF6*, *ARF8*, *ARF10*, *ARF16*, and *ARF17* is focused on rooting phases, not root types, so part of the total gene expression changes could be due to the tissue type used in the analysis.

### 3.3 Expression of nutrition-response miRNAs during T. chinensis root formation

**Figure 4:**
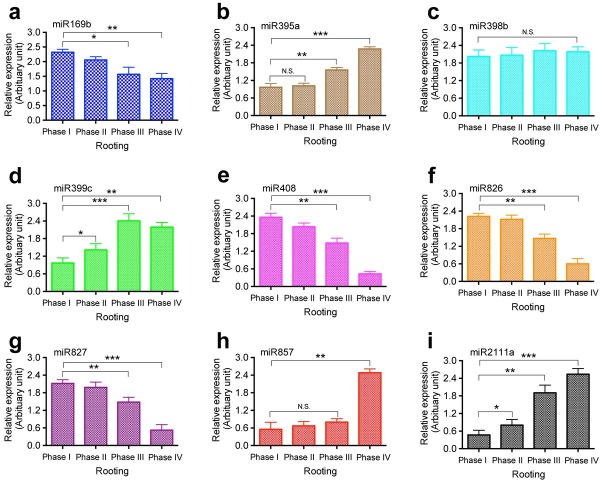
**The involvement of nutrition-response miRNAs in root formation in *T. chinesis***. Nine miRNAs were profiled during in vitro rooting. The miRNAs include miR169b (a), miR395a (b), miR398b (c), miR399c (d), miR408 (e), miR826 (f), miR827 (g), miR857 (h), and miR2111a (i). Total RNA samples were prepared from root tissues of *T. chinensis* harvested at indicated time points and used for qPCR analysis. The *U6* gene was used as an internal reference gene control. Statistically significant differences between groups were determined by one-way ANOVA. Data are presented as means of three independent experiments. Error bars represent standard error. Asterisks indicate significant differences compared to Phase I, as assessed by a *t*-test. *P<0.05, **P<0.01, ***P<0.001, N.S., not statistically significant.

Plants undergo developmental changes due to the availability of nutrients in the soil. Various molecular regulators regulate developmental changes in plants [26, 34]. Some developmental changes are related to the change in miRNA expression [4, 16, 32]. To investigate whether nutrition-response miRNAs participate in root growth and development in *T. chinensis*, we examined the expression levels of nutrition-response miRNAs, including miR169b (Fig. 4a), miR395a (Fig. 4b), miR398b (Fig. 4c), miR399c (Fig. 4d), miR408 (Fig. 4e), miR826 (Fig. 4f), miR827 (Fig. 4g), miR857 (Fig. 4h), and miR2111a (Fig. 4i) in *T. chinensis* root tissues at different rooting phases. The total RNA samples were prepared from root tissues of *T. chinensis* harvested at each time point during the four rooting phases, and a qPCR analysis was performed. The U6 gene was employed as an internal control. Our qPCR results revealed that the expression of miR395a (Fig. 4b), miR399c (Fig. 4d), miR857 (Fig. 4h), and miR2111a (Fig. 4i) was strongly increased at different phases during the rooting process (Fig. 4). MiR395a expression was significantly increased from phase III until phase IV (Fig. 4b). MiR399c expression showed the highest increase at phase III, and the high expression level was maintained until phase IV (Fig. 4d). While miR857 demonstrated the highest expression level at phase IV of rooting (Fig. 4h),

miR2111a was observed to be greatly increased at rooting phase III and reached the highest level at rooting phase IV (Fig. 4i). By contrast, the expression of miR169b (Fig. 4a), miR408 (Fig. 4e), miR826 (Fig. 4f), and miR827 (Fig. 4g) was significantly reduced. Among these four miRNAs, miR408, miR826, and miR827 were detected to gradually decrease during rooting and reached the lowest level at the final phase of rooting, i.e., phase IV (Fig. 4e, f, and g). MiR169b was significantly reduced at rooting phase III (Fig. 4b), and the low expression level persisted until rooting phase IV. Furthermore, our results showed that miR398b (Fig. 4c) remained at a low level of expression throughout the entire rooting process. We only examined the expression of miR169b, miR395a, miR398b, miR399c, miR408, miR826, miR827, miR857, and miR2111a in different rooting phases, not root types, so part of the total changes in expression obtained may be from the tissue type used in the experiments.

### 3.4 Expression of nutrition-response miRNA target genes in T. chinensis roots

**Figure 5:**
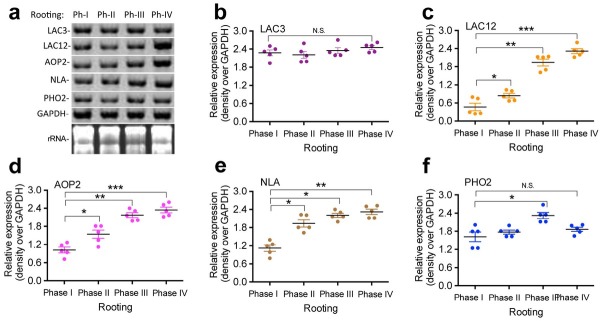
**Expression of nutrition-response miRNA target genes in *T. chinensis* roots**. Total RNA samples were prepared from root tissues of *T. chinensis* harvested at different rooting phases and used for northern blotting analysis (a) of nutrition-related genes *LAC3* (b), *LAC12* (c), *AOP2* (d), *NLA* (e), and *PHO2* (f). Antisense oligonucleotides of *LAC3* (b), *LAC12* (c), *AOP2* (d), *NLA* (e), and *PHO2* (f) were labeled with γ -[32P] ATP and used as probes to detect the transcript levels of these genes. GAPDH rRNA was used as a loading control. Statistically significant differences between groups were determined by one-way ANOVA. Data are presented as means of five independent experiments. Vertical bars indicate standard error. Asterisks indicate significant differences compared to Phase I, as assessed by a *t*-test. *P<0.05, **P<0.01, ***P<0.001, N.S., not statistically significant.

To examine the expression of the nutrition-response miRNA target genes in *T. chinensis* roots, we conducted a northern blot analysis (Fig. 5a) of the nutrition-related

miRNA target genes LAC3 (Fig. 5b), LAC12 (Fig. 5c), AOP2 (Fig. 5d), NLA (Fig. 5e), and PHO2 (Fig. 5f). The total RNAs from the roots of *T. chinensis* harvested at different rooting phases were used for a northern blot analysis. We found that the expression of the nutrition-response miRNA target genes LAC12 (Fig. 5c), AOP2 (Fig. 5d), and NLA (Fig. 5e) were significantly increased in the *T. chinensis* roots at rooting phase II, and these high levels were observed at phases III and IV (Fig. 5). The miRNA target gene PHO2 (Fig. 5f) was significantly increased from rooting phase I until phase III and decreased to the same level observed in phase I by rooting phase IV (Fig. 5f). These results indicate that LAC12 (Fig. 5c), AOP2 (Fig. 5d), and NLA (Fig. 5e) are putative target genes of miR169b (Fig. 4a), miR408 (Fig. 4e), miR826 (Fig. 4f), and miR827 (Fig. 4g), which showed decreased expression levels during the four rooting phases (Fig. 4) in *T. chinensis*. Another nutrition-response miRNA target gene, LAC3, did not show expression level changes throughout rooting (Fig. 5b). We only used roots at different rooting phases to examine expression of LAC3, LAC12, AOP2, NLA, and PHO2. We did not examine different root types, so part of the total changes in expression of the above genes could be due to the tissue type used in the analysis.

## 4 Discussion

Hormone-related genes, nutrition-related genes, transcription factors and transporters are key regulators of plant root growth and development [35, 36, 57]. Additionally, it has been well documented that miRNAs contribute to remarkable changes in root growth and development by modifying the expression of their target genes [36, 53]. For example, in rice, the expression of *OsPHO2* is negatively regulated by miR*399*, which is related to the uptake and translocation of Pi [76], and the expression of *OsAFB2* is negatively regulated by miR393, which is involved in lateral root formation [77]. In *Gossypium hirsutum*, miRNAs regulate the expression of AOP in response to environmental stress [74]. However, no study has investigated the roles of miRNA regulation in root growth and development in woody plants, such as *T. chinensis*. *Taxus chinensis* is highly valuable in the

treatment of various cancers [78, 79, 80, 81]. For the first time, we profiled the differential expression of both phytohormone-response and nutrition-response miRNAs and identified the upregulation and downregulation of miRNAs and their target genes during rooting of *T. chinensis*. In this study, we divided *T. chinensis* rooting into four phases based on the degree of root formation and growth to better identify the relationships between miRNA and root formation. The differential expression of the miRNAs demonstrated that these phases could be differentiated by the ability of nutrition uptake and that the expression of these miRNAs is associated with the physiological responses of the roots in the plants.

Our results showed that the expression of the phytohormone-response miRNAs miR167a, miR171b, and miR391 was significantly decreased from rooting phase II after the formation of the primary root and 1-3 crown roots. Their expression reached the lowest level by rooting phase IV, the final rooting phase in our study that occurs when the primary root grows more than 5 cm with lateral roots and root hairs. These phytohormone-response miRNAs have been shown to regulate their target genes in different plant species [2, 48, 53, 54, 55, 56]. Using a northern blot analysis, we detected the increased expression of ARF6, ARF8, and ARF16. These results suggest that during *T. chinensis* root development, the phytohormone-response miRNAs miR167a, miR171b, and miR391 may play important regulatory roles by decreasing the expression of their target genes, including ARF6, AFR8, and AFR16. Furthermore, we found that four nutrition-response miRNAs were significantly downregulated at rooting phase III; these miRNAs included miR169b, miR408, miR826, and miR827. The expression level of the four miRNAs remained at a low level until rooting phase IV. As previously shown, these nutrition-response miRNAs regulate nutrition uptake by modifying their target genes, including LAC12, AOP2, PHO2, and NLA [16, 17, 77, 82]. Our results showed that the expression of LAC12, AOP2, and NLA increased according to the decreased expression of their regulatory miRNAs from rooting phase III to rooting phase IV. The expression of PHO2 was increased from rooting phase II to rooting phase III. These results suggest that miR169b, miR408, miR826, and miR827 may play very important roles in the regulation of the formation of the crown root and root hair.

By contrast, our results showed that the phytohormone-response miRNAs miR165 and miR319 were significantly upregulated at rooting phase IV when the primary root grew more than 5 cm with both lateral roots and root hairs. In addition, our northern blot analysis demonstrated that the expression of their target gene ARF10 was significantly decreased at rooting phase IV compared with that in rooting phase I. These results indicate that miR165 and miR319 may indirectly contribute to crown root and root hair formation in *T. chinensis* by downregulating the gene expression of their target, i.e., ARF10. In addition, we found that the nutrition-response miRNAs miR399c and miR2111a were significantly increased from rooting phase II throughout phases III and IV and that their target gene PHO2 was significantly decreased accordingly during these rooting phases. Additionally, we found that the nutrition-response miRNA miR395a was upregulated at rooting phase III, and the higher expression level was maintained until phase IV; the nutrition-response miRNA miR857 was significantly increased at rooting phase IV, which is the main phase of lateral root and root hair formation in our study. The function of miR857 is associated with copper homeostasis [72]. These results suggest that the nutrition-response miRNAs miR395a and miR857 and their regulated genes may be necessary for crown root and root hair formation in *T. chinensis*.

In addition to the above up- and downregulated miRNAs, our results revealed that the phytohormone-response miRNAs miR164c, miR170, and miR824 maintained unchanged expression levels throughout rooting. The expression level of their target gene, ARF17, also remained unchanged throughout rooting. In addition, the phytohormone-response miRNA miR398b and its target gene LAC12 exhibited unchanged expression levels throughout rooting. These results suggest that miR164c, miR170, miR824, miR398b and their target genes might not be involved in the regulation of root formation in *T chinensis*.

Taken together, in our current study, we demonstrated that the phytohormone-response miRNAs miR164a, miR165, miR167a, miR171b, miR319, and miR391 and the nutrition-response miRNAs miR169b, miR395a, miR399c, miR408, miR826, miR857, and miR2111a may play a role in the regulation of root formation in *T. chinensis* by modifying their target genes. Among these miRNAs, the phytohormone-response miRNAs miR167a, miR171b, and miR391 may be more important players in the regulation of the initiation of rooting, primary root formation, and the formation of the crown root and root hair by upregulating their target genes’ expression. The nutrition-response miRNAs miR169b, miR408, miR826, and miR827 could be important for regulating crown root and root hair formation by increasing the expression of their target genes. However, the phytohormone-response miRNA miR164a and the nutrition-response miRNAs miR399c and miR2111a may play important roles in the regulation of the initiation of rooting, primary root formation, and the formation of the crown root and root hair by downregulating their target genes’ expression in *T. chinensis*. The phytohormone-response miRNAs miR165 and miR319 and the nutrition-response miRNA miR857 could be important for root hair formation.
